# Effect of an Intersectoral Telemedicine Intervention on Hospitalization in Nursing Home Patients: Specification Curve Analysis of a Stepped Wedge Cluster Randomized Trial

**DOI:** 10.2196/75721

**Published:** 2026-08-19

**Authors:** John Grosser, Sophie Pauge, Birthe Aufenberg, Wolfgang Greiner, Miriam Hertwig, Jenny Unterkofler, David Brücken, Christian Hübel, Optimal@NRW Research Group, Jörg Christian Brokmann, Jörg Christian Brokmann

**Affiliations:** 1 Department of Health Economics and Health Care Management, School of Public Health, Bielefeld University, Universitätsstraße 25, Bielefeld, 33615, Germany, +49 52110686319; 2 Department for Acute and Emergency Medicine, University Hospital RWTH Aachen, Aachen, Germany; 3 Department for Acute and Emergency Medicine, Rhine-Meuse Hospital Würselen, Wüselen, Germany; 4 Optimal@NRW Research Group, Aachen, Germany

**Keywords:** Telemedicine, nursing home, acute care, emergency medical services, specification curve analysis, nursing care, assisted living, early warning system, potentially avoidable hospitalization, hospitalization

## Abstract

**Background:**

The use of emergency medical services in Germany has significantly increased, particularly for nonemergency cases. This issue becomes especially relevant for nursing homes, where staff is confronted with resource shortages and organizational challenges, making emergency medical services involvement a common course of action. This leads to potentially avoidable hospitalizations for nursing home residents and exposes them to severe health risks, such as nosocomial infections. To address this, we developed the Optimal@NRW project, implementing an innovative intersectoral telemedicine intervention that includes an early warning system and mobile nonphysician medical assistants in order to enable outpatient treatment in acute medical cases for nursing home residents. We hypothesize that hospitalizations could be significantly reduced, as patients could be treated on-site in the absence of a life-threatening emergency.

**Objective:**

This study aims to determine the effect of an intersectoral telemedicine-based intervention for acute care situations on hospitalization among residents in nursing homes.

**Methods:**

We conducted a prospective multicenter cluster-randomized stepped wedge trial from May 2021 to April 2023 across 24 nursing homes in western Germany. Nursing homes were randomly assigned to one of four sequences and followed over eight 3-month periods. All sequences crossed stepwise from the control to the intervention phase. We collected primary data in an electronic patient record and claims data from statutory health insurance funds. In a specification curve analysis, we evaluated the intervention effect using multiple mixed-effects regression model specifications with varying modeling approaches, datasets, dependent and independent variables, and operationalizations.

**Results:**

The number of participants ranged from 158 to 285 nursing home residents per sequence and period, totaling 1151 participants in the control phase and 1271 participants in the intervention phase. A total of 1260 regression models were analyzed. Approximately two-thirds of all models exhibited a negative (ie, protective) intervention effect. However, no models exhibited a statistically significant (ie, *P*<.05) intervention effect. The distribution of effect direction and size differed by modeling approach, choice of outcome, and dataset used.

**Conclusions:**

Our results do not support the hypothesis of a significant reduction of hospitalizations among nursing home residents. The effectiveness of the intervention may have been compromised by external factors such as a flood disaster in the study region and the COVID-19 pandemic, which introduced unforeseen challenges. Future studies should prioritize targeted outcomes (potentially avoidable hospitalizations) that align with the specific intervention mechanism, while also allowing for extended implementation periods to ensure that interventions reach their full potential.

## Introduction

The demand for nursing and medical care for nursing home residents (NHR) is steadily increasing due to demographic change and the rising prevalence of chronic diseases, multimorbidity, and care dependency [1]. In Germany, approximately 4.9 million people currently require nursing care, with over half being older than 80 years [2]. Geriatric and multimorbid NHR are significantly sicker compared to older adults living outside such facilities and have higher rates of contact with health care providers [3]. Over the past decade, the use of emergency medical services in Germany has increased substantially [4]. Staffing shortages and organizational challenges in nursing homes, as well as inadequate care capacities in outpatient settings, often result in potentially avoidable hospitalizations for NHR in nonemergency situations, exposing residents to risks like nosocomial infections [5,6], delirium [7,8], and reduced functional capacity [9,10]. This trend is reflected in concepts such as ambulatory care-sensitive conditions (ACSC), core ambulatory care-sensitive conditions (CACSC) [11], and nursing home-sensitive conditions (NHSC) [12], which identify conditions where appropriate on-site or outpatient care could have avoided hospital admission.

These potentially avoidable hospitalizations not only place a financial burden on solidarity-based health care systems [13,14], but also increase costs for municipalities to provide emergency medical services such as ambulances and emergency response vehicles. Furthermore, the growing pressure on nursing homes, driven by societal changes and staff shortages, highlights the need for innovative care models. An intersectoral approach involving all relevant stakeholders is crucial for improving acute care for NHR, as hospital admissions affect not only the nursing homes but also emergency departments, ambulance services, and general practitioners (GPs).

Telemedicine has significant potential to transform acute care by enabling efficient, needs-based medical services across spatial and temporal barriers, optimizing resource use and access, particularly in rural or underserved areas [15,16]. During the COVID-19 pandemic, there was a significant increase in the use of telemedicine in Germany [17]. However, its widespread implementation faces challenges in German nursing homes such as regulatory constraints, reimbursement issues, and limited information technology infrastructure with problems regarding interoperability [18,19]. For instance, it was not compulsory for nursing homes to be connected to the telematics infrastructure until July 2025 (§ 341 (8) of Book V of the German Social Code) [20], which is intended to facilitate secure and efficient data exchange within the health care system. This leads to a fragmented landscape of digitalized medical documentation in nursing homes.

To address these challenges, we designed and implemented an innovative intersectoral telemedicine intervention aimed to enable outpatient treatment for NHR for acute medical conditions. Its effectiveness was evaluated through a prospective multicenter cluster-randomized trial with an open cohort stepped wedge design. The intervention was carried out in 24 nursing homes across the Aachen, Düren, and Heinsberg regions in western Germany, next to the Dutch and Belgian borders, covering both urban and rural areas. In this paper, we analyze the effect of the Optimal@NRW intervention on the hospitalization of NHR through the use of specification curve analysis. In particular, we report the intervention effects from a total of 1260 regression models, resulting from different modeling decisions (eg, choice of dataset, operationalizations, or independent variables).

## Methods

### Reporting

Our methodology follows the CONSORT (Consolidated Standards of Reporting Trials) statement for stepped wedge cluster randomized trials [21]. The checklist can be found in Checklist 1.

### Trial Design

This paper is based on data from a prospective multicenter cluster-randomized trial with an open cohort stepped wedge design [22]. The stepped wedge design was chosen so that all participating nursing homes would eventually receive the intervention, thus fostering commitment to the project. A staggered rollout facilitated the practical and logistical implementation of the intervention. Our open cohort design aimed to recruit a substantial number of participants at the beginning of the trial, while adding more participants during the course of the trial to balance the high expected mortality rates among NHR.

**Figure 1.**
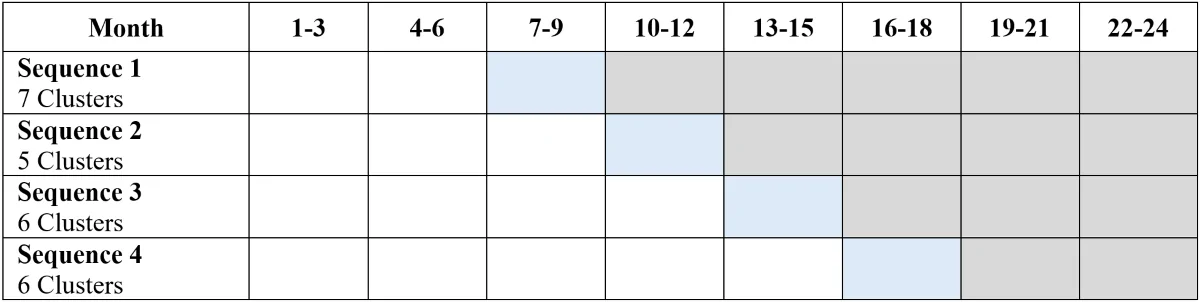
Stepped wedge design of the Optimal@NRW trial. White: control phase, gray: intervention phase, blue: transition phase.

Participants were clustered by nursing home; these clusters were summarized into 4 sequences. All nursing homes started in the control phase, in which participants received standard outpatient care for NHR. Sequences then transitioned stepwise from control to intervention phase, with the timing of the transitions randomized (Figure 1). Each step covered a 3-month interval, and all nursing homes participated in at least 2 intervals (6 mo) in both the control and intervention phases to ensure adequate data collection for each phase. The study took place over 2 years (May 2021 to April 2023), with 8 steps and a total of 24 nursing homes (clusters) across the 4 sequences. The transition phase between the control and intervention phases allowed for staff training and technical setup; data collected during this phase were excluded from our analysis.

### Participants and Data Collection

Potential study participants were included based on the predefined eligibility criteria described in Textbox 1. Recruitment was carried out by the study team within the participating nursing homes (clusters). Following the stepped wedge open cohort study design, participants were enrolled both at the beginning of the study and continuously throughout the study period. All participants gave their written consent before data collection.

Textbox 1. **Inclusion and exclusion criteria of the Optimal@NRW trial.**

**Inclusion criteria:**

- ≥ 18 years of age
- Residence in a participating nursing home
- Signed consent form for study participation and data transfer or consent form signed by carers (for residents who are unable to give consent)

**Exclusion criteria:**

- <18 years of age
- No residence in a participating nursing home
- Nursing home residents in a dependent or employment relationship with a member of the study team
- Lack of clarity about nursing home residents’ capacity to give consent
- Nursing home residents placed in a nursing home by order of the authorities or the courts

We received data on the participants from two sources. First, we used primary data from a study database. The study database contained demographic information on the participants, such as year of birth, sex, and date of death, as well as insurance status and hospitalization data. Primary data were collected by study nurses employed by each nursing home. Second, we received claims data from five statutory health insurance (SHI) funds. The claims data contained demographic information as well as morbidity and hospitalization data. For all study participants who were insured with one of the participating SHI funds, the claims data were provided for the study period as well as an additional 2-year preobservation period. In addition to the information from the study database and claims data, the study nurses also measured the Barthel Index (BI), a patient-reported endpoint to describe the ability for activities of daily living [23], at two time points: at study enrollment (t0) and at the transition to the intervention phase (t1).

However, these two datasets were incompatible with each other for several reasons, with different advantages and disadvantages. First, the primary dataset covered all study participants, while the claims dataset included data only from those participants who were members of one of the participating SHI funds. Second, some information (such as routinely collected morbidity data and data on hospitalizations before the study period) was available only in the claims dataset. Therefore, instead of merging the datasets, we performed the statistical analysis (described below) separately for the primary data and claims data.

### Intervention

The Optimal@NRW project used an innovative, intersectoral intervention to improve acute medical care for residents of nursing homes and residential care facilities (hereafter collectively referred to as “nursing homes”). This intervention consisted of three levels.

First, a telemedical center was initialized at the University Hospital Aachen (UK Aachen) and was available 24/7 for telemedical consultations concerning the NHR. These consultations enabled 3-way communication between patients, nurses, and (tele)physicians. If necessary, mobile nonphysician assistants (nonphysician medical assistants [Nicht-ärztliche Praxisassistent*innen mit Zusatzaufgaben]; NäPa[Z]) were dispatched to perform delegable actions on-site. An electronic health record centralized patient information to ensure informed decision-making and coordination among all care providers.

Second, Optimal@NRW introduced a telemedicine approach via a “virtual hub,” where nursing home staff could call the medical on-call service (116 117) in the event of an acute medical situation involving an NHR. The medical on-call service then assessed the urgency of the situation using a structured initial medical assessment software [24] and determined the next steps. Alternatively, nursing home staff could contact the telephysicians of the UK Aachen directly.

Third, an early warning system (Frühwarnsystem) was implemented, involving routine monitoring of vital signs by nursing home staff, with data transmitted to the telemedicine center for continuous software-based assessment. Further intervention details can be found in the study protocol [25].

During the control phase, care in the participating nursing homes was provided as usual. In acute medical situations, nursing staff were responsible for making decisions independently. This typically involved either contacting a GP or calling the medical on-call service or an ambulance directly.

### Sample Size

For the sample size calculation, we assumed an average of 2.8 days in hospital per patient-quarter based on hospitalization data for German nursing home patients from 2019 [26]. Based on estimates from medical experts at UK Aachen, we hypothesized that the number of days in hospital could be reduced by 30%. Informed by a prior study [27], the intracluster correlation coefficient was set at ρ=0.12. For 24 participating nursing homes, a significance level of α=.05, a power of 1-β=.90, and a SD of 4.5 resulted in a required sample size of 43 residents per nursing home per time point. Considering a 12% quarterly dropout due to high mortality [28] and other factors, the final adjusted sample size was 49 residents per nursing home per time point.

### Randomization

We used stratified randomization at the nursing home level, taking into account bed capacity (small entities with <80 beds and large entities with ≥80 beds) and specialization in dementia care (yes or no) to ensure balanced group allocation. Additionally, block randomization was applied within these stratified groups to avoid imbalances in cluster distribution. The selection of eligible nursing homes was carried out in advance by the study team, while health economists from Bielefeld University, who were not involved in recruitment, data collection, or the design and implementation of the intervention, designed the allocation plan and performed the randomization process to minimize allocation bias. Nursing homes were informed of their assigned sequence by the study team before the start of the trial.

### Objective and Outcomes

The primary objective of our analysis in this paper was to evaluate hospitalization in the intervention versus control phases. It was hypothesized that the intervention would lead to a significant reduction in hospital admissions and, therefore, a significant reduction in days in hospital (it is important to note that the intervention only affects the probability of hospital admission for patients in the nursing home and is not hypothesized to directly affect the length of stay for patients in the hospital).

Hospitalization was measured as a binary variable for each day spent in the nursing home by each patient, defined as 1 if the patient was admitted to the hospital on that day, and 0 otherwise. In addition to a dependent variable considering all hospitalizations, we also defined four further dependent variables, which operationalized potentially avoidable hospitalizations as ACSC hospitalizations, CACSC hospitalizations, NHSC hospitalizations, or same-day discharges (SDD). ACSC refers to a defined set of conditions for the general population in Germany that identifies potentially avoidable hospital admissions. The list is based on *ICD-10* (*International Statistical Classification of Diseases, Tenth Revision*) and includes 40 *ICD-10* diagnoses (ACSC) and a core list of 22 *ICD-10* diagnoses (CACSC) [11]. Additionally, a list of NHSC, comprising 58 *ICD-10* diagnoses that frequently lead to avoidable hospitalizations in this patient cohort, has recently been finalized for Germany [12]. For each hospitalization, we also measured the duration in days (starting at 1 for SDD). All dependent variables available for analysis are shown in Textbox 2

Participant-level characteristics at baseline were summarized by study phase and by allocated sequence. The participant-level characteristics included gender, age, morbidity, and prior hospitalization. For morbidity and prior hospitalization, multiple operationalizations were available. All independent variables available for analysis in the primary and claims datasets are shown in Textbox 3.

Textbox 2. **Available dependent variables. All variables are available at the patient-day level. Variables in the primary dataset were based on information from the study database; variables in the claims dataset were based on information from the statutory health insurance claims data.**

**Available dependent variables**

- Hospitalization: binary variable that equals 1 if there was a hospitalization on the patient-day in question. Equals 0 otherwise.
- Ambulatory care-sensitive conditions (ACSC) hospitalization: binary variable that equals 1 if there was a hospitalization on the patient-day in question and the primary discharge diagnosis of that hospitalization is contained in the ACSC list of *ICD-10* (*International Statistical Classification of Diseases, Tenth Revision*) codes. Equals 0 otherwise.
- Core ambulatory care-sensitive conditions (CACSC) hospitalization: binary variable that equals 1 if there was a hospitalization on the patient-day in question and the primary discharge diagnosis of that hospitalization is contained in the CACSC list of *ICD-10* (*International Statistical Classification of Diseases, Tenth Revision*) codes. Equals 0 otherwise.
- Nursing home-sensitive conditions (NHSC) hospitalization: binary variable that equals 1 if there was a hospitalization on the patient-day in question and the primary discharge diagnosis of that hospitalization is contained in the NHSC list of *ICD-10* (*International Statistical Classification of Diseases, Tenth Revision*) codes. Equals 0 otherwise.
- Same-day discharge (SDD) hospitalization: binary variable, equals 1 if there was a hospitalization on the patient-day in question and the patient in question was discharged on the same day. Equals 0 otherwise.
- Days in hospital: count variable measuring the number of days spent in hospital as the result of hospitalization on the patient-day in question. Equals 0 if there was no hospitalization on the patient-day in question.
- ACSC days in hospital: count variable measuring the number of days spent in hospital as the result of ACSC hospitalization on the patient-day in question. Equals 0 if there was no ACSC hospitalization on the patient-day in question.
- CACSC days in hospital: count variable measuring the number of days spent in hospital as the result of CACSC hospitalization on the patient-day in question. Equals 0 if there was no CACSC hospitalization on the patient-day in question.
- NHSC days in hospital: count variable measuring the number of days spent in hospital as the result of NHSC hospitalization on the patient-day in question. Equals 0 if there was no NHSC hospitalization on the patient-day in question.
- SDD days in hospital: count variable measuring the number of days spent in hospital as the result of SDD hospitalization on the patient-day in question. Equals 0 if there was no SDD hospitalization on the patient-day in question.

Textbox 3. **Available independent variables. All variables are available at the patient-day level. Variables in the primary dataset were based on information from the study database; variables in the claims dataset were based on information from the statutory health insurance claims data.**

**Available independent variables with only one operationalization**

- ID: factor variable, a pseudonym indicating the patient in question; nursing home: factor variable indicating the nursing home of the patient in question.
- Time: factor variable indicating the study quarter (period) of the patient-day in question.
- Study group: binary variable indicating the study phase (control or intervention) of the patient in question on the day in question.
- Gender: factor variable indicating the gender of the patient in question.
- Age: continuous variable obtained by subtracting the birth year of the patient in question from the year of the day in question and scaling around the mean.

**Morbidity (five operationalizations of the morbidity of the patient in question)**

- Factor variable obtained by splitting the most recent observation of the Barthel Index of the patient in question into three categories [29]: no disability (100), moderate disability (56-99), and severe disability (0‐55).
- Factor variable obtained by splitting the most recent observation of the Barthel Index of the patient in question into five categories [30]: independence (100), slight dependence (91-99), moderate dependence (61-90), severe dependence (21-60), and total dependence (0‐20).
- Continuous variable (available only in claims dataset) measuring the Charlson Comorbidity Index of the patient in question, calculated daily based on diagnoses during the 365 days prior to the day in question using the comorbidity package [31].
- Factor variable (available only in claims dataset) obtained by splitting the Charlson Comorbidity Index of the patient in question into 3 categories [29]: no comorbidities (0), moderate comorbidities (1-2), and severe comorbidities (3+).
- Factor variable (available only in claims dataset) measuring the care grade of the patient in question (integer between 0 and 5, assigned by the Health Insurance Medical Service according to § 15 and § 18 of the German Social Code, Book XI (SGB XI)) [32].

**Prior hospitalization (two operationalizations of prior hospitalizations of the patient in question)**

- Binary variable (available only in claims dataset) that equals 1 if the patient in question was admitted to the hospital at least once in the 365 days prior to the day in question. Equals 0 if otherwise.
- Count variable (available only in claims dataset) measuring the number of times the patient in question was admitted to the hospital in the 365 days prior to the day in question.

### Statistical Methods

To estimate the effect of the intervention, we performed mixed-effects regression analysis. However, in specifying our regression model, we faced several modeling decisions that offered no clear correct choice. Instead of fitting only a single regression model, we addressed these issues by performing a specification curve analysis. In such an analysis, researchers report the results of all “reasonable specifications,” defined as the set of model specifications that are “(1) sensible tests of the research question, (2) expected to be statistically valid and (3) not redundant with other specifications in the set” [33]. The estimated effect sizes from each model are then ordered by size and visualized in a diagram–the specification curve. Specification curve analysis can be considered a formal extension of the common practice of considering the robustness of results to model specification decisions [33]. For our analysis, we ran a total of 1260 separate regression models with different combinations of modeling approaches, datasets, dependent and independent variables, and operationalizations.

First, we needed to choose which modeling approach to use. For this, we considered three options: (1) a negative binomial count model for the number of hospital days at the patient-quarter level, (2) a negative binomial count model for the number of hospitalizations at the patient-quarter level, and (3) a binomial model for hospital admission at the patient-day level. For the count models, we used a log link function. For the binomial regression, we used a complementary log-log (*cloglog*) link function. Unlike logit or probit link functions, *cloglog* link functions are asymmetrical and are therefore most appropriate when the probability of an event is very high or very low [34-36]. This was the case for our data, as there was no hospitalization on the vast majority of person-days in both datasets.

Second, as the primary data collected by the study team and the claims data provided by the SHI funds were incompatible, we needed to choose on which data to fit the regression model. Third, after choosing a dataset, we needed to choose how to operationalize the outcome: as measuring all, ACSC, CACSC, NHSC, or SDD hospitalizations/days in hospital. For ease of reference, we refer to models using ACSC hospitalizations as the outcome as “ACSC models” (and so on for the remaining operationalizations).

In each model specification, we included the variables study group (the treatment variable)*,* time (to model secular trends)*,* nursing home, and id (to model cluster- and patient-level measurement using random intercepts). For the first and second modeling approaches (count models for the number of hospital days and the number of hospitalizations at the patient-quarter level), we also included a factor variable controlling for the time spent in the nursing home by each patient in a given quarter. However, we still needed to decide which combination of the remaining available independent variables (gender, age, morbidity, and prior hospitalization) to use as control variables.

Finally, multiple operationalizations were available for two independent variables in one or both datasets, with no clear theoretical basis for choosing between these operationalizations. In particular, the primary dataset included two possible operationalizations of morbidity, while the claims dataset included five possible operationalizations of morbidity and two possible operationalizations of prior hospitalization (recall Textbox 3). For each modeling approach, all possible combinations of outcome and control variables amounted to a total of 5*2*2*3=60 model specifications on the primary dataset and 5*2*2*6*3=360 model specifications on the claims dataset. This yields a total of 420 model specifications per modeling approach and 1260 model specifications across all 3 modeling approaches. For each of these model specifications, we removed all patients who had missing values in at least one variable that was included in the model specification. The model formulas for each modeling approach on both datasets are illustrated in Textbox 4.

Textbox 4. **Model formulas on the primary and claims datasets.**

**Primary dataset**

1. negative binomial regression (patient-quarter level): hospital days (5 options)~study group+time+gender (2 options)+age (2 options)+morbidity (3 options)+nursing home (random intercept)+id (random intercept)
2. negative binomial regression (patient-quarter level): hospital admissions (5 options)~ study group+ time+gender (2 options)+age (2 options)+morbidity (3 options)+nursing home (random intercept)+id (random intercept)
3. cloglog regression (patient-day level): any hospitalization (5 options)~study group+time+gender (2 options)+age (2 options)+morbidity (3 options)+nursing home (random intercept)+id (random intercept)

For each modeling approach on the primary dataset: 5*2*2*3=60 possible formulas.(Note: for covariates, noninclusion is an option).

**Claims dataset**

1. negative binomial regression (patient-quarter level): hospital days (5 options)~study group+time+gender (2 options)+age (2 options)+morbidity (6 options)+prior hospitalization (3 options)+nursing home (random intercept)+id (random intercept)
2. negative binomial regression (patient-quarter level): hospital admissions (5 options)~study group+time+gender (2 options)+age (2 options)+morbidity (6 options)+prior hospitalization (3 options)+nursing home (random intercept)+id (random intercept)
3. cloglog regression (patient-day level): any hospitalization (5 options)~study group+time+gender (2 options)+age (2 options)+morbidity (6 options)+prior hospitalization (3 options)+nursing home (random intercept)+id (random intercept)

For each modeling approach on the claims dataset: 5*2*2*6*3=360 possible formulas. (Note: for covariates, noninclusion is an option).

For the first and second modeling approach (count models for the number of hospital days and the number of hospitalizations at the patient-quarter level), we calculated rate ratios by exponentiating the regression coefficients for the study group variable. Note that the rate ratios in the first modeling approach refer to the number of hospital days attributable to hospitalizations in a given quarter, not the number of hospital days that took place during the quarter.

The regression coefficients of the *cloglog* models in the third modeling approach do not allow for a straightforward interpretation in terms of log odds or odds ratios. Therefore, for the third modeling approach, we calculated the intervention effect on the average participant in terms of the yearly probability of hospitalization. This was determined by calculating the predicted daily (total, ACSC, CACSC, NHSC, or SDD) hospitalization probability in the control and intervention phases for a hypothetical participant with modal or mean values for all variables and subtracting the former from the latter (random effects were disregarded for this calculation). We then multiplied the resulting value by 365 to yield the predicted difference in additional (total, ACSC, CACSC, NHSC, or SDD) hospitalizations per 365 days spent in the nursing home between the intervention and control phases.

We processed the data and ran the regression models in the RStudio (Posit Software, PBC) environment using the tidyverse [37], comorbidity [31], and glmmTMB [38] packages and visualized the model configurations (choice of dataset, control variables, and variable operationalizations) and resulting intervention effects for each modeling approach in a specification curve using the specr package [39]. For each modeling approach, we also calculated the number and share of intervention effects by direction, the mean, minimum, and maximum size of the intervention effect, and the median, minimum, and maximum *P* value across all models.

### Ethical Considerations

This study was approved by the Ethics Committee of the UK Aachen (CTC-A No. 19-019; EK 463/20). All participants provided informed consent before data collection, which covered both the primary and secondary datasets. The study adhered to the ethical principles outlined in the Declaration of Helsinki.

Primary data for the study were collected by study nurses in the participating nursing homes and stored in a study database, in accordance with the ethics committee approval. Claims data were received from participating SHI funds after authorization by the relevant bodies, including the Federal Office for Social Security (Bundesamt für Soziale Sicherung) and state-level supervisory offices, in accordance with German law. Both datasets were transmitted and stored in accordance with the European General Data Protection Regulation. Detailed descriptions of data security, storage, and transfer mechanisms, as well as further information on the legal foundation of data collection and use, can be found in the study protocol [25]. The study was registered May 10, 2021, under ClinicalTrials.gov (NCT04879537). A study protocol was published September 27, 2022, in Trials following the SPIRIT checklist [25].

## Results

### Recruitment and Participant Flow

**Figure 2.**
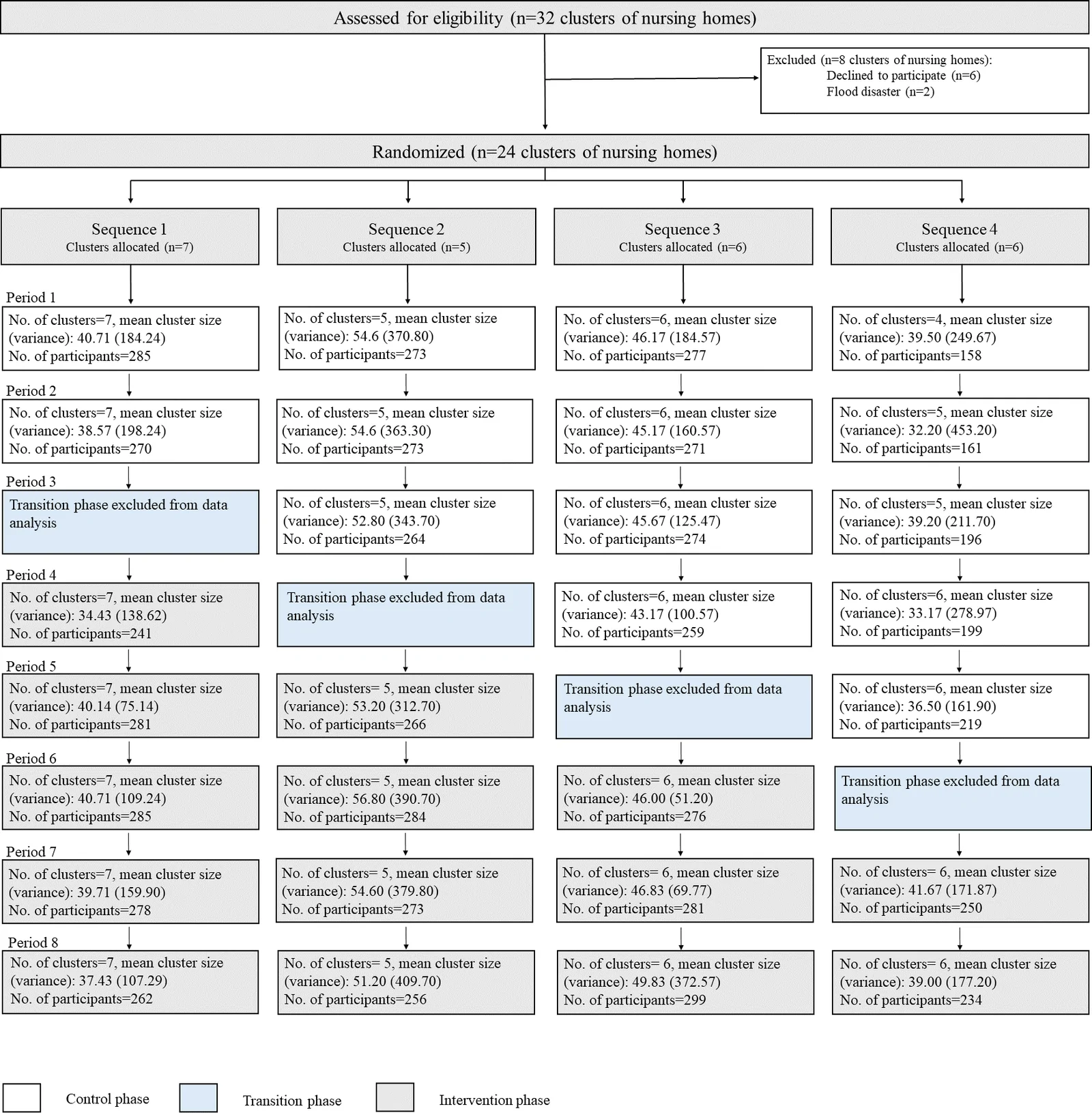
Study flowchart of participants and nursing homes.

Figure 2 shows the participant flowchart, which illustrates the recruitment, randomization, and progression of clusters and participants through the study phases of our stepped wedge cluster randomized trial. Initially, 32 clusters of nursing homes were assessed for eligibility. Six nursing homes declined to participate, while 2 nursing homes had to cancel their planned participation after randomization had taken place due to a flood disaster in their region, which forced those homes to shut down operations temporarily. Therefore, 2 additional nursing homes were recruited for sequence 4 in periods 2 and 3. In total, 24 nursing homes participated (in four sequences containing between 4 and 7 nursing homes each). The number of participants assessed varied across periods, ranging from 158 to 285 individuals per sequence per period. Data from the transition phase was not used in our analysis and is therefore excluded from the flowchart.

### Baseline Characteristics

The baseline characteristics of participants in the control and intervention phases are shown in Table 1. In both the primary and claims datasets, there were more patients, more patient-days, and a slightly lower percentage of female participants in the intervention compared to the control phase. The phases were well balanced in terms of mean age of participants and mean BI (in the BI subgroup). In the claims dataset, participants in both phases also had a similar mean Charlson Comorbidity Index (CCI), median care grade, and number of prior hospitalizations (in the past year). The BI scores indicate that the participants are highly dependent on assistance in their daily lives, while the CCI scores indicate a high level of comorbidity.

As our study followed an open cohort stepped wedge design, some patients were enrolled in both the control and intervention phases, while others were enrolled only in the control phase or only in the intervention phase. As recommended by the CONSORT extension for stepped wedge trials [21], we therefore also summarized participants’ baseline characteristics by allocated sequence in Table 2.

**Table 1.** Baseline characteristics by study phase in the primary and claims datasets.

Variable	Control	Intervention
Primary dataset		
Total patients, N	1151	1271
Total patient-days, N	281,484	306,947
Age (years^a^), mean (95% CI)	81.02 (80.35-81.68)	81.32 (80.7-81.94)
Female, n (%)	769 (66.81)	826 (64.99)
BI^a,b,c^mean (95% CI)	50.37 (48.49-52.26)	50.85 (49.11-52.58)
Claims dataset		
Total patients, N	762	858
Total patient-days, N	185,329	211,213
Age (years^a^), mean (95% CI)	80.14 (79.3‐80.98)	80.66 (79.9‐81.43)
Female, n (%)	510 (66.93)	552 (64.34)
BI^a,c,^^d^ mean (95% CI)	51.88 (49.53-54.22)	52.19 (50.08-54.29)
CCI^a,^^e^ mean (95% CI)	3.10 (2.95-3.26)	2.97 (2.83-3.11)
PG^a^^,^^f^^,g^ median	4	4
Hospitalizations in past year^a^*,* mean (95% CI)	1.28 (1.16-1.4)	1.25 (1.13-1.37)

a At enrollment and/or the start of the intervention phase.

b Data available for subgroup only! (n=941 control phase, n=1157 intervention phase).

c BI: Barthel Index.

d Data available for subgroup only! (n=632 control phase, n=780 intervention phase).

e CCI: Charlson Comorbidity Index.

f Data available for subgroup only! (n=725 control phase, n=769 intervention phase).

g PG: care grade (German: Pflegegrad).

**Table 2.** Baseline characteristics by allocated sequence in the primary and claims datasets.

Variable	SEQ^a^ 1	SEQ 2	SEQ 3	SEQ 4	Total
Primary dataset					
Total patients, N	421	379	431	323	1554
Total patient-days, N	155,088	158,191	159,145	116,007	588,431
Age (years^b^), mean (95% CI)	82.94 (81.99-83.88)	80.47 (79.22-81.73)	82.55 (81.6-83.48)	78.51 (77.17-79.85)	81.31 (80.75-81.86)
Female, n (%)	278 (66.03)	256 (67.55)	276 (64.04)	206 (63.78)	1016 (65.38)
BI^b^^,^^c^^,^^d^, mean (95% CI)	52.43 (49.36-55.51)	51.20 (48.05-54.36)	47.14 (44.18-50.1)	54.60 (50.91‐58.3)	51.07 (49.47‐52.67)
Claims dataset					
Total patients, N	294	257	278	202	1031
Total patient-days, N	111,280	108,591	100,796	75,875	396,542
Age (years^b^), mean (95% CI)	82.62 (81.48‐83.76)	79.32 (77.75‐80.88)	81.60 (80.37‐82.83)	77.78 (76.09‐79.46)	80.57 (79.88‐81.27)
Female, n (%)	199 (67.69)	165 (64.20)	175 (62.95)	131 (64.85)	670 (64.99)
BI^b^^,^^d^^,^^e^, mean (95% CI)	52.94 (49.32‐56.56)	52.33 (48.38‐56.28)	47.93 (44.23‐51.63)	56.56 (51.9‐61.22)	52.09 (50.12‐54.06)
CCI^b^^,^^g^, mean (95% CI)	2.93 (2.7‐3.16)	3.17 (2.89‐3.44)	2.93 (2.69‐3.17)	2.97 (2.66‐3.28)	3.00 (2.87‐3.13)
PG^b^^,^^f^^,^^h^*,* median	4	3	4	4	4
Hospitalizations in past year^b^*,* mean (95% CI)	1.34 (1.17‐1.52)	1.50 (1.24‐1.75)	1.26 (1.05‐1.48)	1.59 (1.31‐1.87)	1.41 (1.3‐1.52)

a SEQ: Sequence.

b At enrollment.

c Data available for subgroup only! (n=941 control phase, n=1157 intervention phase).

d BI: Barthel Index.

e Data available subgroup only! (n=632 control phase, n=780 intervention phase).

f Data available subgroup only! (n=725 control phase, n=769 intervention phase).

g CCI: Charlson Comorbidity Index.

h PG: care grade (German: Pflegegrad).

### Outcome Measures

The primary outcomes of our analysis were the number of hospitalizations and days spent in hospital. A descriptive comparison of outcomes by study phase is provided in Table 3. As for the baseline characteristics, we also present the number of hospitalizations and days in hospital by allocated sequence in Table 4. Notably, although the claims dataset covers fewer patients than the primary dataset, the number of ACSC and CACSC hospitalizations (both total and per patient-year in the nursing home) is higher in the claims than in the primary dataset for each of the four sequences. Furthermore, the number of NHSC hospitalizations per patient-year is also larger in the claims dataset than in the primary dataset.

**Table 3.** Hospitalizations and days in hospital by study phase in the primary and claims datasets.

Variable	Control	Intervention
Primary dataset		
Total hospitalizations, n	893	1005
Total ACSC^a^ hospitalizations, n	215	213
Total CACSC^b^ hospitalizations, n	162	164
Total NHSC^c^ hospitalizations, n	310	302
Total SDD^d^ hospitalizations, n	306	368
Hospitalizations per patient-year in NH^e^, mean	1.16	1.20
ACSC hospitalizations per patient-year in NH, mean	0.28	0.25
CACSC hospitalizations per patient-year in NH, mean	0.21	0.20
NHSC hospitalizations per patient-year in NH, mean	0.40	0.36
SDD hospitalizations per patient-year in NH, mean	0.40	0.44
Total days in hospital, n	6070	5838
Total ACSC days in hospital, n	1831	1382
Total CACSC days in hospital, n	1491	1229
Total NHSC days in hospital, n	1960	1430
Total SDD days in hospital, n	306	368
Days in hospital per patient, mean	5.27	4.59
ACSC days in hospital per patient, mean	1.59	1.09
CACSC days in hospital per patient, mean	1.30	0.97
NHSC days in hospital per patient, mean	1.70	1.13
SDD days in hospital per patient, mean	0.27	0.29
Claims dataset		
Total hospitalizations, n	615	641
Total ACSC hospitalizations, n	267	278
Total CACSC hospitalizations, n	256	263
Total NHSC hospitalizations, n	248	260
Total SDD hospitalizations, n	126	105
Hospitalizations per patient-year in NH, mean	1.21	1.11
ACSC hospitalizations per patient-year in NH, mean	0.53	0.48
CACSC hospitalizations per patient-year in NH, mean	0.50	0.45
NHSC hospitalizations per patient-year in NH, mean	0.49	0.45
SDD hospitalizations per patient-year in NH, mean	0.25	0.18
Total days in hospital, n	6106	5775
Total ACSC days in hospital, n	1570	1917
Total CACSC days in hospital, n	1512	1848
Total NHSC days in hospital, n	1414	1766
Total SDD days in hospital, n	126	105
Days in hospital per patient, mean	8.01	6.73
ACSC days in hospital per patient, mean	2.06	2.23
CACSC days in hospital per patient, mean	1.98	2.15
NHSC days in hospital per patient, mean	1.86	2.06
SDD days in hospital per patient, mean	0.17	0.12

a ACSC: Ambulatory care-sensitive condition.

b CACSC: Core ambulatory care-sensitive condition.

c NHSC: Nursing home-sensitive condition.

d SDD: Same-day discharges.

e NH: Nursing home.

**Table 4.** Hospitalizations and days in hospital by allocated sequence in the primary and claims datasets.

Variable	SEQ^a^ 1	SEQ 2	SEQ 3	SEQ 4	Total
Primary dataset					
Total hospitalizations, n	498	481	496	423	1898
Total ACSC^b^ hospitalizations, n	131	124	83	90	428
Total CACSC^c^ hospitalizations, n	95	96	63	72	326
Total NHSC^d^ hospitalizations, n	171	152	176	113	612
Total SDD^e^ hospitalizations, n	177	136	204	157	674
Hospitalizations per patient-year in NH^f^, mean	1.17	1.11	1.14	1.33	1.18
ACSC hospitalizations per patient-year in NH, mean	0.31	0.29	0.19	0.28	0.27
CACSC hospitalizations per patient-year in NH, mean	0.22	0.22	0.14	0.23	0.20
NHSC hospitalizations per patient-year in NH, mean	0.40	0.35	0.40	0.36	0.38
SDD hospitalizations per patient-year in NH, mean	0.42	0.31	0.47	0.49	0.42
Total days in hospital, n	3198	3093	2812	2802	11,905
Total ACSC days in hospital, n	896	839	623	855	3213
Total CACSC days in hospital, n	764	669	495	792	2720
Total NHSC days in hospital, n	912	939	809	730	3390
Total SDD days in hospital, n	177	136	204	157	674
Days in hospital per patient, mean	7.60	8.16	6.52	8.67	7.66
ACSC days in hospital per patient, mean	2.13	2.21	1.45	2.65	2.07
CACSC days in hospital per patient, mean	1.81	1.77	1.15	2.45	1.75
NHSC days in hospital per patient, mean	2.17	2.48	1.88	2.26	2.18
SDD days in hospital per patient, mean	0.42	0.36	0.47	0.49	0.43
Claims dataset					
Total hospitalizations, n	338	380	264	274	1256
Total ACSC hospitalizations, n	154	144	118	129	545
Total CACSC hospitalizations, n	147	134	113	125	519
Total NHSC hospitalizations, n	147	128	109	124	508
Total SDD hospitalizations, n	67	57	36	71	231
Hospitalizations per patient-year in NH, mean	1.11	1.28	0.96	1.32	1.16
ACSC hospitalizations per patient-year in NH, mean	0.51	0.48	0.43	0.62	0.50
CACSC hospitalizations per patient-year in NH, mean	0.48	0.45	0.41	0.60	0.48
NHSC hospitalizations per patient-year in NH, mean	0.48	0.43	0.39	0.60	0.47
SDD hospitalizations per patient-year in NH, mean	0.22	0.19	0.13	0.34	0.21
Total days in hospital, n	2889	3896	2553	2570	11,881
Total ACSC days in hospital, n	1065	774	795	853	3487
Total CACSC days in hospital, n	1032	723	766	839	3360
Total NHSC days in hospital, n	986	674	701	819	3180
Total SDD days in hospital, n	67	57	36	71	231
Days in hospital per patient, mean	9.83	15.05	9.18	12.72	11.52
ACSC days in hospital per patient, mean	3.62	3.01	2.86	4.22	3.38
CACSC days in hospital per patient, mean	3.51	2.81	2.76	4.15	3.26
NHSC days in hospital per patient, mean	3.35	2.62	2.52	4.05	3.08
SDD days in hospital per patient, mean	0.23	0.22	0.13	0.35	0.22

a SEQ: sequence.

b ACSC: Ambulatory care-sensitive condition.

c CACSC: Core ambulatory care-sensitive condition.

d NHSC: Nursing home-sensitive condition.

e SDD: Same-day discharges.

f NH: nursing home.

The same holds for days in hospital: the number of ACSC and CACSC days in hospital (both total and per patient) is higher in the claims than in the primary dataset, as is the number of NHSC days in hospital per patient. Notably, despite the claims dataset containing 643 fewer total hospitalizations than the primary dataset, it contained only 24 fewer days in hospital. Overall, these results indicate substantial differences in the quantity and quality of hospitalization data between the primary and claims datasets (ie, incomplete hospitalization information in the primary dataset), necessitating a separate analysis for each dataset.

### Specification Curve Analysis

#### Modeling Approach 1: Count Models for Days in Hospital

**Figure 3.**
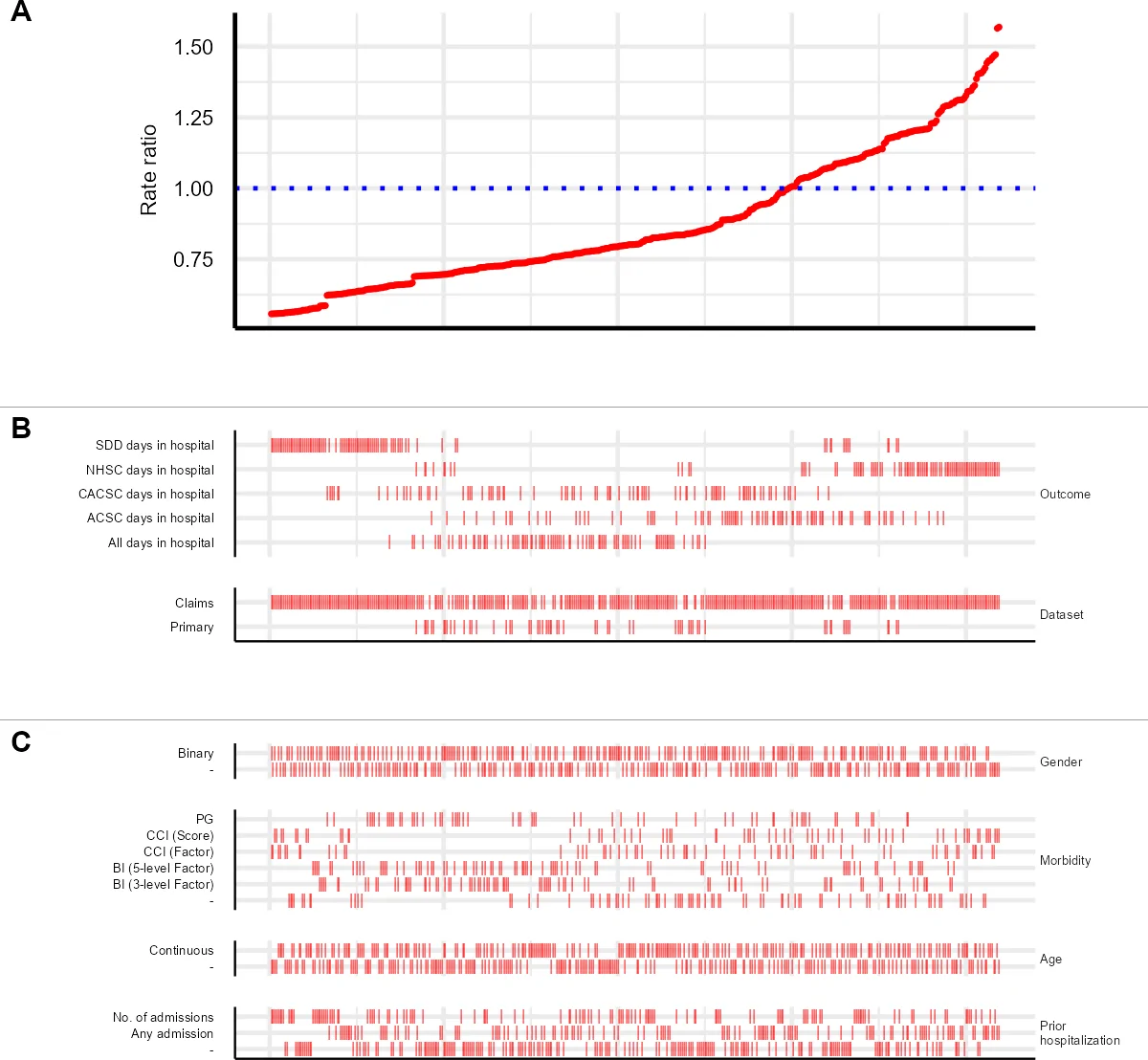
Specification curve for modeling approach 1. SDD: same-day discharges; NHSC: nursing home-sensitive conditions; CACSC: core ambulatory care-sensitive conditions; ACSC: ambulatory care-sensitive conditions; PG: care grade (German: Pflegegrad); CCI: Charlson Comorbidity Index; BI: Barthel Index.

For modeling approach 1, we ran a total of 420 count regression models with days in hospital as the dependent variable. Of these, one model failed to converge, leaving 419 models for analysis. To illustrate the effect of our modeling choices on the size and direction of the intervention effect, Figure 3 shows the specification curve for the 419 model specifications of modeling approach 1.

The specification curve reveals that the operationalization of the outcome variable has the most noticeable influence on the size of the intervention effect. In particular, SDD models had the lowest rate ratios, while NHSC models had the largest rate ratios. The remaining model specification choices did not have as clear a systematic effect on the size of the intervention effect.

Table 5 shows the proportion of negative intervention effects, the mean, minimum, and maximum effect sizes as well as the median, minimum, and maximum *P* values for the 419 regression models: overall, by outcome variable, and by dataset. Over 71% (298/419) of regression models found a negative (ie, protective) intervention effect, with a mean rate ratio of 0.88. This intervention effect was nonsignificant for all 419 regression models, with a median *P* value of .54 (IQR .34-.75) (although some SDD models exhibited *P* values as low as .08).

NHSC models had the lowest proportion of negative effects (12/84, or less than 15%). Effect sizes (rate ratios) ranged from 0.56 to 1.57, with a mean rate ratio of 0.88. Models using the primary dataset had a higher proportion of negative effects (and a slightly lower mean rate ratio) than those using the claims dataset.

**Table 5.** Effect direction, size and significance for modeling approach 1 by outcome variable.

Outcome variable (days in hospital)	Negative effects	RR^a^, mean (range)	*P* value, median (range)
Overall (N=419), n (%)	298, 71.12	0.88 (0.56-1.57)	.54 (.08->.99)
By outcome variable (days in hospital), n (%)			
All (n=84)	84 (100.00)	0.76 (0.66-0.85)	.37 (.19-.56)
ACSC^b^ (n=84)	51 (60.71)	0.95 (0.69-1.29)	.82 (.48-.99)
CACSC^c^ (n=83)	79 (95.18)	0.80 (0.62-1.07)	.66 (.38->.99)
NHSC^d^ (n=84)	12 (14.29)	1.18 (0.69-1.57)	.65 (.36-.95)
SDD^e^ (n=84)	72 (85.71)	0.68 (0.56-1.18)	.20 (.08-.78)
By dataset, n (%)			
Primary (n=59)	47 (79.66)	0.83 (0.69-1.18)	.53 (.26-.78)
Claims (n=360)	251 (69.72)	0.88 (0.56-1.57)	.55 (.08->.99)

a RR: Relative risk.

b ACSC: Ambulatory care-sensitive condition.

c CACSC: Core ambulatory care-sensitive condition.

d NHSC: Nursing home-sensitive condition.

e SDD: Same-day discharges.

#### Modeling Approach 2: Count Models for Hospitalizations

**Figure 4.**
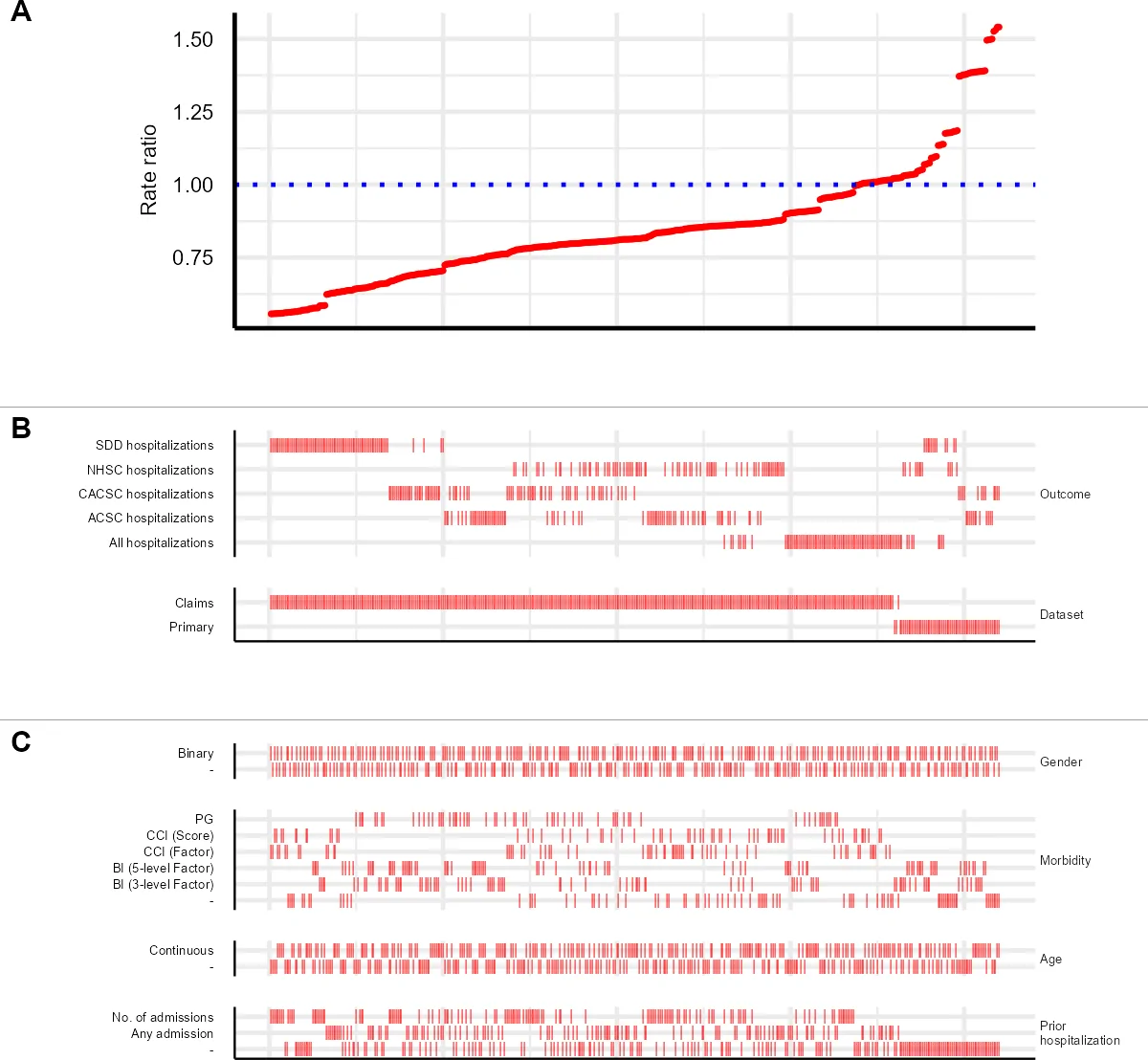
Specification curve for modeling approach 2. SDD: same-day discharges; NHSC: nursing home-sensitive conditions; CACSC: core ambulatory care-sensitive conditions; ACSC: ambulatory care-sensitive conditions; PG: care grade (German: Pflegegrad); CCI: Charlson Comorbidity Index; BI: Barthel Index.

For modeling approach 2, we ran a total of 420 count regression models with the number of hospitalizations as the response. Of these models, none failed to converge, leaving 420 models for analysis. Figure 4 shows the specification curve for these 420 model specifications.

The specification curve reveals that the choice of dataset and the operationalization of the outcome variable have the most noticeable influence on the size of the intervention effect. In particular, models using the primary dataset and SDD models had larger rate ratios than those using the claims dataset or other outcome operationalizations. Within the claims dataset, models using all hospitalizations as the outcome had larger rate ratios than those using other outcome operationalizations. The remaining model specification choices did not have as clear a systematic effect on the size of the intervention effect.

Table 6 shows the proportion of negative intervention effects, the mean, minimum, and maximum effect sizes as well as the median, minimum, and maximum *P* values for the 420 regression models: overall, by outcome variable, and by dataset. Over 80% (339/420) of regression models found a negative (ie, protective) intervention effect, with a mean rate ratio of 0.85. As for modeling approach 1, the intervention effect was nonsignificant for all 420 regression models, with a median *P* value of .42 (IQR .26-.57; although some SDD models exhibited *P* values as low as .08).

Models using all hospitalizations as the outcome had the lowest proportion of negative effects at 60.71% (51/84). Effect sizes (rate ratios) ranged from 0.56 to 1.54, with a mean rate ratio of 0.85. Table 6 also confirms the notable difference in the proportion of negative effects and mean rate ratio between the primary and the claims datasets.

**Table 6.** Effect direction, size, and significance for modeling approach 2 by outcome and dataset.

Outcome variable (days in hospital)	Negative effects	RR^a^, mean (range)	*P* value, median (range)
Overall (N=420), n (%)	339 (80.71)	0.85 (0.56-1.54)	.42 (.08-.99)
By outcome (days in hospital) (n=84), n (%)			
All	51 (60.71)	0.97 (0.86-1.14)	.82 (.36-.99)
ACSC^b^	72 (85.71)	0.89 (0.72-1.50)	.40 (.12-.57)
CACSC^c^	72 (85.71)	0.84 (0.67-1.54)	.28 (.13-.42)
NHSC^d^	72 (85.71)	0.87 (0.77-1.19)	.51 (.39-.90)
SDD^e^	72 (85.71)	0.68 (0.56-1.18)	.20 (.08-.78)
By dataset, n (%)			
Primary (n=60)	0 (0.00)	1.22 (1.02-1.54)	.45 (.12-.90)
Claims (n=360)	339 (94.17)	0.79 (0.56-1.02)	.41 (.08-.99)

a RR: Relative risk.

b ACSC: Ambulatory care-sensitive condition.

c CACSC: Core ambulatory care-sensitive condition.

d NHSC: Nursing home-sensitive condition.

e SDD: Same-day discharges.

#### Modeling Approach 3: Binary Models for Hospitalization

**Figure 5.**
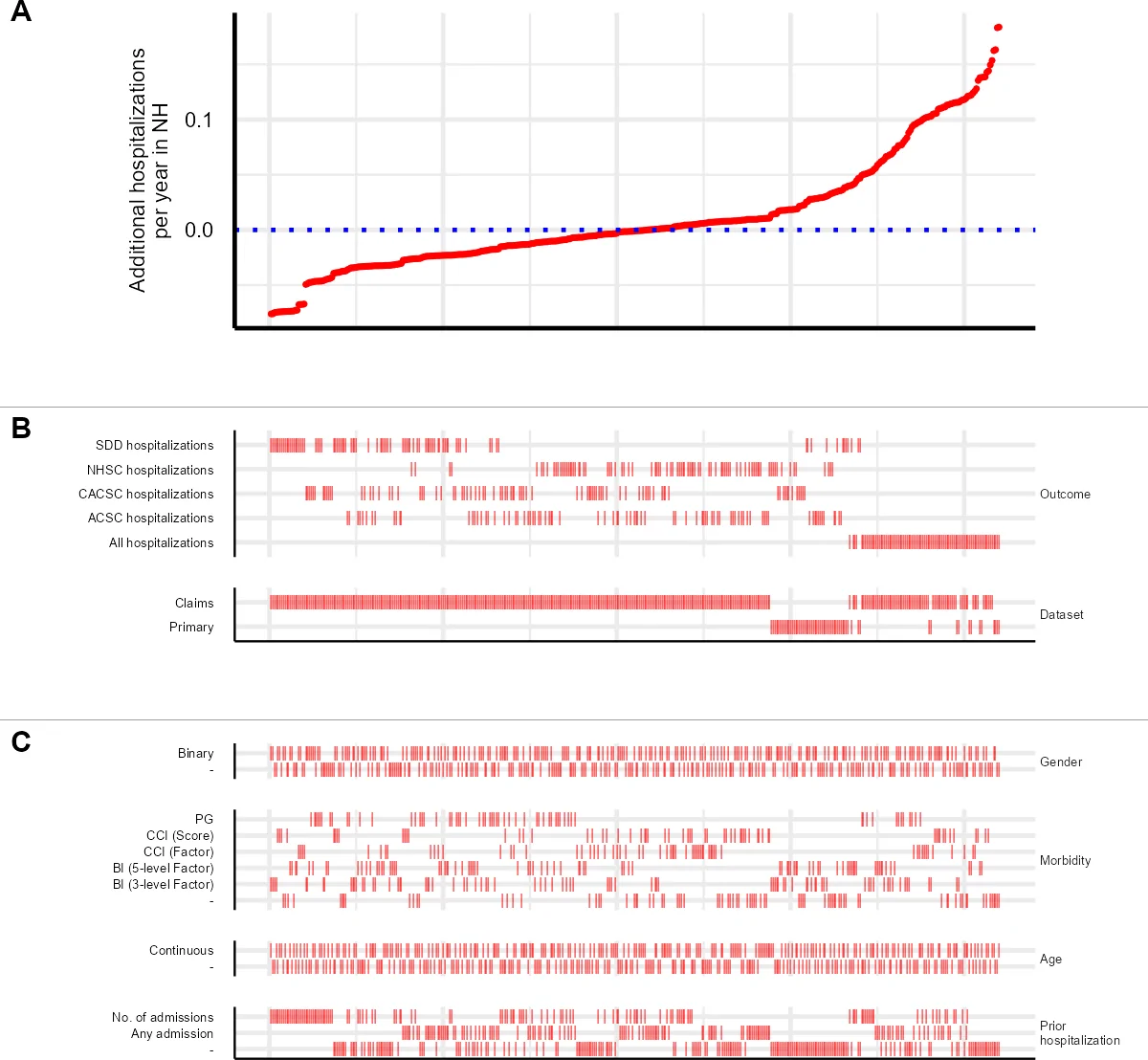
Specification curve for modeling approach 3. SDD: same-day discharges; NHSC: nursing home-sensitive conditions; CACSC: core ambulatory care-sensitive conditions; ACSC: ambulatory care-sensitive conditions; PG: care grade (German: Pflegegrad); CCI: Charlson Comorbidity Index; BI: Barthel Index

For modeling approach 3, we ran a total of 420 binomial regression models with daily hospitalization as the (binary) response. Of these models, none failed to converge, leaving 420 models for analysis. Figure 5 shows the specification curve for these 420 model specifications.

As for modeling approach 2, the specification curve reveals that the choice of dataset and the operationalization of the outcome variable have the most noticeable influence on the size of the intervention effect. In particular, models using all hospitalizations had a larger number of additional hospitalizations per 365 days spent in the nursing home than those using other outcome operationalizations. Furthermore, among those models using outcome operationalizations other than all hospitalizations, those using the primary dataset had a larger number of additional hospitalizations per 365 days spent in the nursing home than those using the claims dataset. The remaining model specification choices did not have as clear a systematic effect on the size of the intervention effect.

Table 7 shows the proportion of negative intervention effects, the mean, minimum, and maximum effect sizes as well as the median, minimum, and maximum *P* values for the 420 regression models. Over half of the regression models (217/420, 51.67%) found a negative (ie, protective) intervention effect. Once again, the intervention effect was nonsignificant for all 420 regression models, with a median *P* value of .56 (IQR .28-.81; although some models exhibited *P* values as low as .07).

**Table 7.** Effect direction, size, and significance for modeling approach 3 by outcome and dataset.

Outcome variable (days in hospital)		Negative effects	Effect, mean (range)	*P* value, median (range)
Overall (N=420), n (%)		217 (51.67)	0.01 (–0.08 to 0.18)	.56 (.07->.99)
By outcome (days in hospital) (n=84), n (%)				
All		0 (0.00)	0.10 (0.04 to 0.18)	.37 (.07-.76)
ACSC^a^		48 (57.14)	0.00 (–0.04 to 0.04)	.78 (.09->.99)
CACSC^b^		65 (77.38)	–0.01 (–0.05 to 0.02)	.58 (.10->.99)
NHSC^c^		32 (38.10)	0.00 (–0.03 to 0.03)	.84 (.30-.99)
SDD^d^		72 (85.71)	–0.03 (–0.08 to 0.05)	.22 (.12-.62)
By dataset, n (%)				
Primary (n=60)		0 (0.00)	0.05 (0.01 to 0.18)	.26 (.07-.72)
Claims (n=360)		217 (60.28)	0.00 (–0.08 to 0.15)	.62 (.12->.99)

a ACSC: Ambulatory care-sensitive condition.

b CACSC: Core ambulatory care-sensitive condition.

c NHSC: Nursing home-sensitive condition.

d SDD: Same-day discharges.

Models with all hospitalizations as the outcome had the lowest proportion of negative effects, as all such models found a positive (ie, nonprotective) intervention effect. Effect sizes (the number of additional hospitalizations per 365 days spent in the nursing home) ranged from −0.08 to 0.18, with a mean of 0.01. This indicates that the absolute size of most intervention effects was not clinically relevant. As for modeling approach 2, zero of the 60 models on the primary dataset found a negative intervention effect, although the proportion of negative effects in the claims dataset was lower than for modeling approach 2.

## Discussion

### Principal Findings

We investigated the effect of an intersectoral telemedicine intervention on hospitalization probability for residents in nursing homes. Our analysis was based on data from a multicenter, prospective, cluster randomized controlled trial with an open cohort stepped wedge design. We conducted a specification curve analysis, for which we considered 1260 different regression model specifications, of which 1259 converged. Of these, none found a statistically significant (ie, *P*<.05) intervention effect.

While 854 of 1259 converged models found a (nonsignificant) negative intervention effect (67.83%), the distribution of these effects differed by modeling approach, choice of outcome, and dataset used. For modeling approaches 1 and 2 (count models for the number of hospital d and the number of hospitalizations at the patient-quarter level), more than 70% (298/419) and more than 80% (339/420) of the models found a negative (ie, protective) intervention effect, respectively. For modeling approach 3 (binary models for hospitalization probability at the patient-d level), only just over 50% (217/420) of models found a negative intervention effect. The differences in effect direction by choice of outcome and dataset were even more pronounced. While only 135 of 252 models using all hospitalizations (53.57%) and 116 of 252 NHSC models (46.03%) found a negative intervention effect, these proportions rose to 216 of 252 SDD models (85.71%) and 216 of 251 CACSC models (86.06%). And while almost three-quarters of models (807/1080) on the claims dataset found a negative intervention effect, only 26.26% (47/179) of models on the primary dataset did so.

Based on these results, our analysis does not support the hypothesis of a significant negative intervention effect. This may be due to either (1) issues with our data or methodology, (2) external factors such as the flood disaster and the COVID-19 pandemic, or (3) the actual noneffectiveness of the intervention.

Concerning (1), it could be argued that operationalizing the outcome using SDD or CACSC hospitalizations (where the proportion of negative intervention effects is also higher) may be more appropriate than using all hospitalizations, since the Optimal@NRW intervention specifically targeted potentially avoidable hospitalizations and included no mechanism to prevent necessary hospitalizations. Furthermore, the claims dataset contains more total ACSC and CACSC hospitalizations than the primary dataset, even though the primary dataset contains about 1.5 times as many patients and patient-days. This indicates that there may have been a data quality issue in the primary dataset (see limitations). Therefore, one could argue that using the claims dataset (where the proportion of negative intervention effects is also higher) is more appropriate than using the primary dataset.

Restricting the space of model specifications according to this line of argument can only increase the proportion of negative intervention effects. It does not change the fact that the intervention effect was nonsignificant in all models. However, since the sample size calculation was conducted for all hospitalizations (rather than one of the operationalizations of potentially avoidable hospitalizations), our study might not have had a sufficient sample size to reliably detect intervention effects for potentially avoidable hospitalizations. This indicates that future studies explicitly focused on, and powered for the analysis of, potentially avoidable hospitalizations (and using high-quality datasets) may be better equipped to identify a (true) significant intervention effect.

Our results were also affected by the flood disaster and the COVID-19 pandemic (see limitations). These external factors directly impacted operations in the participating nursing homes, which might have affected the number of hospitalizations (and potentially avoidable hospitalizations, in particular). For instance, studies showed that there were fewer hospital admissions in general during the COVID-19 pandemic [13,14] in Germany. The potential impact of COVID-19 restrictions at the outset of our study may have led to more avoided hospitalizations compared to later phases, complicating the distinction between external influences and the intervention’s true effect. This indicates that future studies conducted without the influence of these external factors may reach a different result than our analysis.

However, the possibility remains that our results reflect the true noneffectiveness of the intervention. This would be surprising, as several international studies have demonstrated an effect of telemedicine in nursing homes reducing hospital admissions (particularly emergency admissions) [40,41]. For instance, Joseph et al (2020) [41] found that NHR receiving telemedicine in acute care situations were less likely to be transferred to emergency departments than a control group.

In Germany, a number of pilot projects [42,43] have emerged that align with our objectives by integrating telemedicine solutions to enhance acute care management in order to reduce potentially avoidable hospitalizations. A previous study, conducted in the same region as the Optimal@NRW project, evaluated a tele-emergency medical service, in which a remote emergency physician supported paramedics to reduce the need for on-site physician involvement [42]. In a randomized controlled trial, the researchers demonstrated that 58% of patients in the intervention group could be dispatched solely by paramedics, compared to only 6% in the control group. This finding underlines the feasibility and suitability of telemedicine approaches in acute care situations and highlights that the successful implementation of telemedicine approaches requires a significant adaptation period before reaching full operational efficiency [42]. The nursing homes in our study exhibited an increasing use of teleconsultations over time. It therefore remains questionable for our study whether an intervention phase of 6 to 15 months was sufficient to fully realize the intervention’s potential and achieve sustained effectiveness. Moreover, our intervention did not focus exclusively on a telemedicine approach for acute care situations but also integrated mobile nonphysician medical assistants, whom telephysicians could dispatch to expand the range of on-site treatments in nursing homes. Previous pilot studies have applied similar approaches, such as the community paramedic system (Gemeindenotfallsanitäter), which aimed to provide on-site medical assessment and care for low-acuity emergencies. Studying the Gemeindenotfallsanitäter, Seeger et al [43] demonstrated that 60% of NHR were treated on-site. While they concluded that Gemeindenotfallsanitäter saves resources of emergency services and hospitals, it is essential to note that their study primarily focused on process outcomes rather than quantifying patient-relevant clinical endpoints, as we did.

Successful implementation of telemedicine and complex interventions also strongly depends on the motivation of affected staff. Alongside our study, we systematically monitored the acceptance of participating nursing staff to gain deeper insights into the dynamics, facilitators, and barriers influencing the effective implementation of our intervention. While the results, which have been published elsewhere [44], suggested increased telemedicine acceptance during the intervention phase, these differences did not reach statistical significance. This underlines the need for an extended implementation period to foster greater acceptance over time. The numbers of teleconsultations also differed greatly between nursing homes, in that 15 nursing homes used 75% of all teleconsultations. This cannot be purely attributed to less acute care situations, but also to nursing home specific reasons, which require further research.

Rather than focusing purely on telemedicine to enhance acute care situations, we advocate for a holistic, intersectoral approach that extends beyond merely addressing acute medical needs at the point of care. Therefore, for the Optimal@NRW intervention, we chose a comprehensive approach that integrates preventive measures via our Frühwarnsystem to identify potential health risks in advance and incorporates a follow-up strategy through mobile nonphysician medical assistants to enhance on-site treatment opportunities. However, implementing multiple inherently complex components at once may overwhelm practical application due to staff shortages and workload consolidation in the German health care system. For instance, the Frühwarnsystem measurement was intended to be conducted daily by the nursing staff, but proved to be impractical. Instead, simpler and more manageable solutions are needed, such as implementing wearable technology for NHR, which does not require additional staff resources [45].

We also anticipated incorporating an intersectoral approach by engaging various stakeholders involved in acute care situations, particularly GPs. Recent qualitative and quantitative research in the German nursing sector indicates that including GPs in telemedical and digital interventions for NHR should increase access to GPs outside regular consultation hours, save time, and ease the workload of communicating with GPs for nursing home staff [46,47]. Meanwhile, initial evidence from pilot and pre-post intervention studies in Germany suggests that GP-based telemedicine interventions for NHR can reduce avoidable hospitalizations [48,49], although more evidence from high-quality randomized control trials is needed. Telemedicine interventions for NHR have also been found to reduce avoidable hospitalizations in other contexts, including France [50,51] and the United States [52].

However, during our study, we realized that including GPs proved particularly challenging, with only a very limited number ultimately participating. We invested significant time and effort into personal outreach (such as phone calls and meetings) and provided financial incentives; however, these efforts proved ineffective at recruiting GPs. Given the critical role of GPs in coordinating and providing medical care for NHR, their inclusion in future intersectoral interventions is essential. This underscores the need for a more effective outreach strategy to enhance GP engagement and facilitate their integration into telemedicine-based acute care models.

### Strengths and Limitations

To our knowledge, this is the first study evaluating a comprehensive telemedicine-based approach to treat acute care situations of NHR in Germany regarding patient-relevant clinical outcomes. The main strength of our research is the use of specification curve analysis, which allowed us to investigate the impact of different modeling choices on the direction, size, and significance of the intervention effect. It ensures the robustness of the presented results.

A methodological limitation of our approach is that our specification curve analysis is mainly descriptive. Inferential specification curve analysis is also possible and even recommended in the literature, with Simonsohn et al (2020) suggesting test statistics that include the median effect across all specifications (in our case, 0.11 additional hospitalizations per y for an average participant) and the share of specifications with significant effects in the predicted direction (in our case, none) [33]. To obtain the distributions of these test statistics, they propose modifying the data by shuffling the columns containing randomly assigned variables. For this shuffled dataset, the null hypothesis is now known to be true. Calculating the test statistic of interest for hundreds of random samples from the modified dataset then delivers the estimated distribution of the test statistic under the null hypothesis [33].

In this paper, we did not undertake an inferential specification curve analysis. This was mainly due to the structure of our data. Each row in our datasets represented a patient-day. The assignment of these patient-days to a treatment phase (intervention or control) depends on the date of the patient-day and the nursing home in which the patient was recruited. Therefore, shuffling the treatment column as described above would be inappropriate. Furthermore, even if a suitably shuffled dataset could be created, fitting 1260 mixed-effects regression models for hundreds of samples would have exceeded available computational resources. However, the lack of an inferential specification curve analysis does not represent an overly significant limitation of our results, as all intervention effects were nonsignificant.

Although the primary data was continuously checked for plausibility by a data monitoring team throughout the study, it can be assumed that there are inconsistencies in the primary dataset due to the self-documentation process and an initially paper-based procedure. This became particularly evident when compared with the SHI claims data where, for example, the number of ACSC hospitalizations per patient-year was approximately twice as high in the claims dataset as in the primary dataset. Such inconsistencies raise concerns about the internal validity of the primary dataset. Moreover, models using the (quality-assured) claims data exhibited a notably higher proportion of negative intervention effects. However, this finding must be interpreted with caution, as the smaller sample size in the claims dataset may introduce bias, potentially overestimating the observed intervention effects even though no model showed significant results. It must also be noted that relevant confounders of morbidity included in our regression models were either not captured in the primary dataset (eg, care grade) or could only be applied for a subsample of included NHR due to missing data (eg, BI).

Another limitation of this study is the assumed effect size of a 30% reduction in hospital days during the intervention phase, which served as the basis for the sample size calculation. In retrospect, this estimate appears overly optimistic, potentially leading to an underpowered study design. Furthermore, the heterogeneous utilization of intervention components across different nursing homes may have contributed to variations in the intervention’s effectiveness, further complicating the ability to detect a statistically significant effect. This variability, combined with the potential underestimation of the required sample size, likely impacted the study’s ability to demonstrate a significant effect, particularly when considering general hospitalization outcomes rather than specific, targeted endpoints.

Accordingly, future studies should base their sample size calculations on small-to-moderate effect sizes. Although this would increase the sample size needed, larger sample sizes may be more feasible in future projects using only claims data, since researchers would not need to collect primary data for each participant. This may also reduce dropout rates, keeping the total sample size needed manageable. Furthermore, future studies may be able to shorten the transition phase (in which participants are recruited and data is collected, but not analyzed), which would further reduce the total sample size needed. Finally, using only claims data, rather than both primary and claims data, may alleviate the concerns regarding primary data quality outlined above.

Beyond these methodological concerns, our study faced several external limitations that may have affected our data and results. A significant portion of the data collection period was impacted by the COVID-19 pandemic and the June 2021 flood disaster. These events affected participant numbers, the feasibility of the study, and data collection processes, while also influencing the analysis, as they could not be directly accounted for. Such unforeseen events may limit the generalizability of our findings.

### Conclusion

Based on the results of our specification curve analysis, we conclude that the Optimal@NRW intervention had no significant effect on hospitalization probability for NHR. This could be attributed either to (1) methodological limitations of our study and analysis, (2) external factors, such as the flood disaster and COVID-19 pandemic, or (3) the actual noneffectiveness of the intervention. To address (1), future research should choose more targeted outcomes measuring potentially avoidable hospitalizations, such as SDD or CACSC, which align more closely with the intended underlying mechanisms of the intervention. To address (3), holistic, intersectoral, telemedicine-based interventions should prioritize optimizing usability for intervention components to ensure seamless integration into existing workflows, while minimizing additional workload for nursing staff. Moreover, future studies could benefit from the progressive scalability of telemedicine-based treatments, leveraging potential upscaling scenarios that enhance both feasibility and efficiency. As familiarity and confidence in telemedicine approaches continue to grow, particularly in acute care settings, increasing trust in digital health care solutions may further strengthen their impact on patient-relevant endpoints, and long-term sustainability.

## Supplementary material

10.2196/75721 Checklist 1 CONSORT Checklist for Stepped Wedge Trials.

## Abbreviations

ACSC: ambulatory care-sensitive condition
BI: Barthel Index
CACSC: core ambulatory care-sensitive condition
CCI: Charlson Comorbidity Index
CONSORT: Consolidated Standards of Reporting Trials
GP: general practitioner
*ICD-10*: *International Classification of Diseases, 10th Revision*
NHR: nursing home residents
NHSC: nursing home-sensitive condition
SDD: same-day discharge
SHI: statutory health insurance
UK Aachen: University Hospital Aachen

## References

[1] Schaeffer D, Hämel K, Kriwy P, Jungbauer-Gans M (2020). Handbook of Medical Sociology.

[2] Schwinger A, Kuhlmey A, Greß S, Klauber J, Jacobs K, Behrendt S (2024). Nursing Report 2024: Arrival of the Baby Boomers – Challenges for Care [Book in German].

[3] (2024). Sachverständigenrat Zur Begutachtung Der Entwicklung Im Gesundheitswesen Und In Der Pflege. Nachhaltiger Einsatz einer knappen Ressource - Gutachten 2024 - Fachkräfte im Gesundheitswesen. PUBLISSO.

[4] Schehadat MS, Scherer G, Groneberg DA, Kaps M, Bendels MHK (2021). Outpatient care in acute and prehospital emergency medicine by emergency medical and patient transport service over a 10-year period: a retrospective study based on dispatch data from a German emergency medical dispatch centre (OFF-RESCUE). BMC Emerg Med.

[5] Saviteer SM, Samsa GP, Rutala WA (1988). Nosocomial infections in the elderly. Am J Med.

[6] Isigi SS, Parsa AD, Alasqah I, Mahmud I, Kabir R (2023). Predisposing factors of nosocomial infections in hospitalized patients in the United Kingdom: systematic review. JMIR Public Health Surveill.

[7] Siddiqi N, House AO, Holmes JD (2006). Occurrence and outcome of delirium in medical in-patients: a systematic literature review. Age Ageing.

[8] Ormseth CH, LaHue SC, Oldham MA, Josephson SA, Whitaker E, Douglas VC (2023). Predisposing and precipitating factors associated with delirium: a systematic review. JAMA Netw Open.

[9] Gill TM, Allore HG, Holford TR, Guo Z (2004). Hospitalization, restricted activity, and the development of disability among older persons. JAMA.

[10] Loyd C, Markland AD, Zhang Y (2020). Prevalence of hospital-associated disability in older adults: a meta-analysis. J Am Med Dir Assoc.

[11] Sundmacher L, Fischbach D, Schuettig W, Naumann C, Augustin U, Faisst C (2015). Which hospitalisations are ambulatory care-sensitive, to what degree, and how could the rates be reduced? Results of a group consensus study in Germany. Health Policy.

[12] Valk-Draad MP, Gröne O, Schult T, Stahl K, Bohnet-Joschko S (2023). Needs-based care for nursing home residents through nursing home-sensitive hospital cases: final report [Report in German]. https://innovationsfonds.g-ba.de/downloads/beschluss-dokumente/434/2023-08-16_PSK_Ergebnisbericht.pdf.

[13] Weeks WB, Ventelou B, Paraponaris A (2016). Rates of admission for ambulatory care sensitive conditions in France in 2009-2010: trends, geographic variation, costs, and an international comparison. Eur J Health Econ.

[14] Shinotsuka M, Matsumura S, Okada T (2020). Emergency admissions of ambulatory care sensitive conditions at a Japanese local hospital: an observational study. J of Gen and Family Med.

[15] Selvaraj S (2024). Enhancing healthcare access in rural communities: assessing the influence of telehealth services and information technology. Int J Sci Res.

[16] Beckers R, Telepflege SV, Marx G, Rossaint R, Marx N (2021). Telemedicine: Foundations and Practical Application in Inpatient and Outpatient Facilities [Book in German].

[17] Knörr V, Dini L, Gunkel S (2022). Use of telemedicine in the outpatient sector during the COVID-19 pandemic: a cross-sectional survey of German physicians. BMC Prim Care.

[18] Kitschke L, Traulsen P, Waschkau A, Steinhäuser J (2024). Determinants of the implementation of telemedicine in nursing homes: a qualitative analysis from Schleswig-Holstein [Article in German]. Z Evid Fortbild Qual Gesundhwes.

[19] Dassel K, Busch A, Lutze M (2024). Reducing workload through nursing software: which quality criteria matter? [Report in German].

[20] (1988). Sozialgesetzbuch (SGB) Fünftes Buch (V) – Gesetzliche Krankenversicherung.

[21] Hemming K, Taljaard M, McKenzie JE (2018). Reporting of stepped wedge cluster randomised trials: extension of the CONSORT 2010 statement with explanation and elaboration. BMJ.

[22] Hemming K, Haines TP, Chilton PJ, Girling AJ, Lilford RJ (2015). The stepped wedge cluster randomised trial: rationale, design, analysis, and reporting. BMJ.

[23] Mahoney FI, Barthel DW (1965). Functional evaluation: the Barthel Index. Md State Med J.

[24] Koech L, Ströhl S, Lauerer M (2024). Redirection of patients from the emergency department to ambulatory care: a feasibility study [Article in German]. Gesundheitswesen.

[25] Brücken D, Unterkofler J, Pauge S (2022). Optimal@NRW: optimized acute care of nursing home residents using an intersectoral telemedical cooperation network - study protocol for a stepped-wedge trial. Trials.

[26] Jacobs K, Kuhlmey A, Greß S, Klauber J, Schwinger A (2019). Care Report 2019: More Personnel in Long-Term Care – But From Where?.

[27] Hoffmann F, Schmiemann G (2017). Influence of age and sex on hospitalization of nursing home residents: a cross-sectional study from Germany. BMC Health Serv Res.

[28] Vossius C, Selbæk G, Šaltytė Benth J, Bergh S (2018). Mortality in nursing home residents: a longitudinal study over three years. PLoS One.

[29] Morishima T, Sato A, Nakata K (2021). Barthel Index-based functional status as a prognostic factor in young and middle-aged adults with newly diagnosed gastric, colorectal and lung cancer: a multicentre retrospective cohort study. BMJ Open.

[30] Shah S, Vanclay F, Cooper B (1989). Improving the sensitivity of the Barthel Index for stroke rehabilitation. J Clin Epidemiol.

[31] Gasparini A (2018). comorbidity: an R package for computing comorbidity scores. J Open Source Softw.

[32] (1994). Elftes Buch Sozialgesetzbuch (SGB XI) – Soziale Pflegeversicherung.

[33] Simonsohn U, Simmons JP, Nelson LD (2020). Specification curve analysis. Nat Hum Behav.

[34] Prasetyo RB, Kuswanto H, Iriawan N, Ulama BSS A comparison of some link functions for binomial regression models with application to school drop-out rates in East Java.

[35] Alves JSB, Bazán JL, Arellano-Valle RB (2023). Flexible cloglog links for binomial regression models as an alternative for imbalanced medical data. Biom J.

[36] Kitaw TA, Tilahun BD, Abate BB, Haile RN (2024). Minimum acceptable diet and its predictors among children aged 6-23 months in Ethiopia. A multilevel cloglog regression analysis. Matern Child Nutr.

[37] Wickham H, Averick M, Bryan J (2019). Welcome to the Tidyverse. J Open Source Softw.

[38] Brooks M, Kristensen K, Benthem K (2017). glmmTMB balances speed and flexibility among packages for zero-inflated generalized linear mixed modeling. R J.

[39] Masur PK, Scharkow M (2023). Conducting and visualizing specification curve analyses.

[40] Groom LL, McCarthy MM, Stimpfel AW, Brody AA (2021). Telemedicine and telehealth in nursing homes: an integrative review. J Am Med Dir Assoc.

[41] Joseph JW, Kennedy M, Nathanson LA, Wardlow L, Crowley C, Stuck A (2020). Reducing emergency department transfers from skilled nursing facilities through an emergency physician telemedicine service. West J Emerg Med.

[42] Schröder H, Beckers SK, Borgs C (2024). Long-term effects of a prehospital telemedicine system on structural and process quality indicators of an emergency medical service. Sci Rep.

[43] Seeger I, Günther U, Schmiemann G, Hoffmann F (2022). Care of older patients by community emergency paramedics: comparison of community-dwellers and nursing home residents [Article in German]. Med Klin Intensivmed Notfmed.

[44] Offermann J, Ziefle M, Optimal@NRW Research Group (2024). You are not alone! Care professionals’ acceptance of telemedicine in nursing homes comparing pre- and post-implementation evaluations. Electronics (Basel).

[45] Frech J, Jackson D, Naber J, Masukevitch A, King T Nursing home patient monitoring system for improved healthcare and outcomes.

[46] Martin T, Veldeman S, Großmann H, Fuchs-Frohnhofen P, Czaplik M, Follmann A (2024). Long-term adoption of televisits in nursing homes during the COVID-19 crisis and following up Into the postpandemic setting: mixed methods study. JMIR Aging.

[47] Denny K, Gundlack J, Bay J (2026). The GP’s offices are far away and the gain would be great.’-a qualitative study on nursing home staff’s perspectives on digital solutions for communication issues with general practitioners. Scand J Prim Health Care.

[48] Ohligs M, Stocklassa S, Rossaint R, Czaplik M, Follmann A (2020). Employment of telemedicine in nursing homes: clinical requirement analysis, system development and first test results. Clin Interv Aging.

[49] Redeker AC, Martin T, Veldeman S (2025). Impact of regular televisits on unplanned hospital admissions of nursing home residents in rural Germany: a pre-post intervention study. BMC Geriatr.

[50] Marjanovic N, Jonchier M, Guenezan J, Delelis-Fanien H, Reuter PG, Mimoz O (2024). Telemedicine in nursing home residents requiring a call to an emergency medical communication center. J Am Med Dir Assoc.

[51] Gayot C, Laubarie-Mouret C, Zarca K (2022). Effectiveness and cost-effectiveness of a telemedicine programme for preventing unplanned hospitalisations of older adults living in nursing homes: the GERONTACCESS cluster randomized clinical trial. BMC Geriatr.

[52] Grabowski DC, O’Malley AJ (2014). Use of telemedicine can reduce hospitalizations of nursing home residents and generate savings for medicare. Health Aff (Millwood).

